# Accuracy of Dolphin visual treatment objective (VTO) prediction software on class III patients treated with maxillary advancement and mandibular setback

**DOI:** 10.1186/s40510-016-0132-2

**Published:** 2016-06-17

**Authors:** Robert J. Peterman, Shuying Jiang, Rene Johe, Padma M. Mukherjee

**Affiliations:** Department of Orthodontics, Rutgers School of Dental Medicine, 110 Bergen Street, Newark, NJ 07103 USA; Department of Institutional Assessment and Quality Improvement, Rutgers School of Dental Medicine, 110 Bergen Street, Newark, NJ 07103 USA

## Abstract

**Background:**

Dolphin® visual treatment objective (VTO) prediction software is routinely utilized by orthodontists during the treatment planning of orthognathic cases to help predict post-surgical soft tissue changes. Although surgical soft tissue prediction is considered to be a vital tool, its accuracy is not well understood in tow-jaw surgical procedures. The objective of this study was to quantify the accuracy of Dolphin Imaging’s VTO soft tissue prediction software on class III patients treated with maxillary advancement and mandibular setback and to validate the efficacy of the software in such complex cases.

**Methods:**

This retrospective study analyzed the records of 14 patients treated with comprehensive orthodontics in conjunction with two-jaw orthognathic surgery. Pre- and post-treatment radiographs were traced and superimposed to determine the actual skeletal movements achieved in surgery. This information was then used to simulate surgery in the software and generate a final soft tissue patient profile prediction. Prediction images were then compared to the actual post-treatment profile photos to determine differences.

**Results:**

Dolphin Imaging’s software was determined to be accurate within an error range of +/− 2 mm in the *X*-axis at most landmarks. The lower lip predictions were most inaccurate.

**Conclusions:**

Clinically, the observed error suggests that the VTO may be used for demonstration and communication with a patient or consulting practitioner. However, Dolphin should not be useful for precise treatment planning of surgical movements. This program should be used with caution to prevent unrealistic patient expectations and dissatisfaction.

## Background

One of the great challenges in orthodontics is the treatment planning and management of orthognathic surgical cases. These cases require a combination of both orthodontics and orthognathic surgery to achieve a well-balanced occlusion, proper function, and harmonious facial esthetics. Depending on the type of skeletal imbalance, oral and maxillofacial surgeons perform orthognathic surgeries involving the maxilla and/or the mandible for these patients.

Orthodontics, esthetics, and visual aids for soft tissue prediction have progressed since the early 1970s from the use of acetate tracing paper to computer-based line drawings to more modern technologies in the 1990s where computers could alter patient photographs in an attempt to predict surgical outcomes [1–5]. Visual treatment objective (VTO) images help the orthodontist to predict hard and soft tissue changes that may occur as a result of surgery and can be utilized to treatment plan orthognathic cases and to communicate with patients and surgeons. Seventy percent of prospective orthognathic surgery patients mention esthetics as their principle motivation, further highlighting the importance of soft tissue treatment planning [6].

It is the doctor’s legal, moral, and ethical responsibility to inform the patient of the risks versus benefits, options of camouflage versus surgery, and treatment versus no treatment [7]. However, VTOs might lead to unrealistic patient expectations causing dissatisfaction with post-surgical results [8]. This is a valid concern, as the errors of the prediction in two-jaw surgical cases are not well understood.

Current literature is controversial with regards to the level of accuracy of the soft tissue predictions, and which areas of the face are best predicted by these software programs for two-jaw orthognathic cases [9]. Therefore, the primary objective of this study is to quantify the accuracy of Dolphin Imaging’s VTO soft tissue prediction software on patients treated with maxillary advancement and mandibular setback.

## Methods

### Patient sample

After receiving IRB approval, records from the Rutgers University School of Dental Medicine, Departments of Orthodontics and Oral and Maxillofacial Surgery were reviewed. The inclusion criteria included non-growing patients, cervical vertebral maturation (CVM) stage 5; treated with comprehensive orthodontic treatment and orthognathic surgery involving both maxillary advancement and/or mandibular setback [10]. We excluded subjects with craniofacial anomalies, syndromes, history of trauma, patients who underwent any other surgical procedures, or poor quality records. Fourteen subjects (11 females and 3 males; mean age of 22.55; standard deviation 4.5714) were selected and determined to have complete records. All patients were treated with 0.022 × 0.028-in. pre-adjusted edgewise orthodontic appliances.

### Cephalometric analysis

**Fig. 1:**
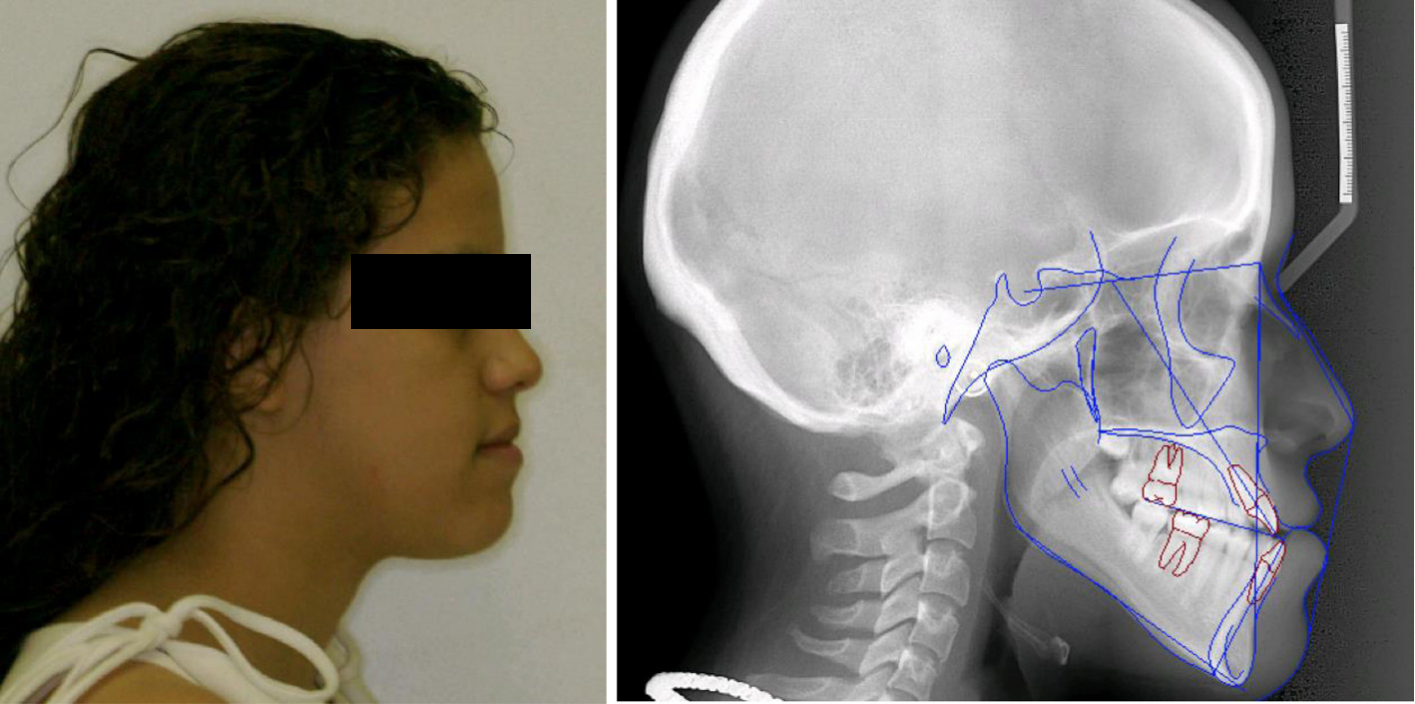
Illustration of pre-treatment profile photograph and traced cephalometric radiograph of a subject included in the sample

**Fig. 2:**
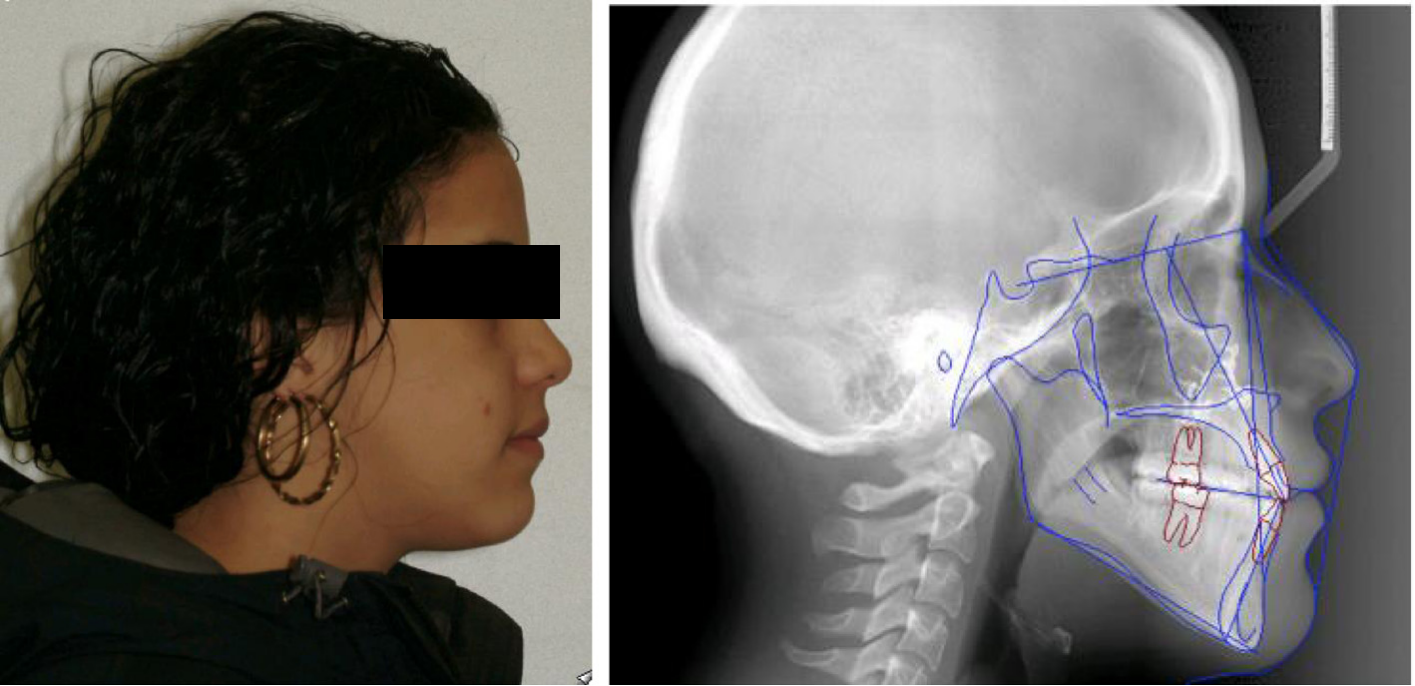
Illustration of post-treatment profile photograph and traced cephalometric radiograph of a subject included in the sample

**Fig. 3:**
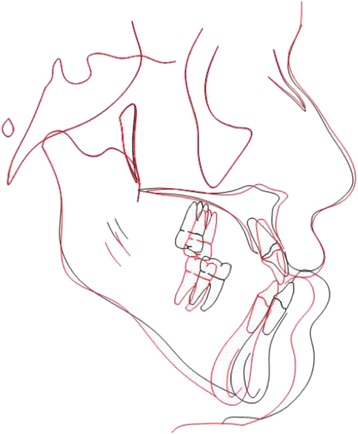
Superimposition of pre- and post-treatment and traced cephalometric tracing showing advancement of the maxilla and setback of the mandible

**Fig. 4:**
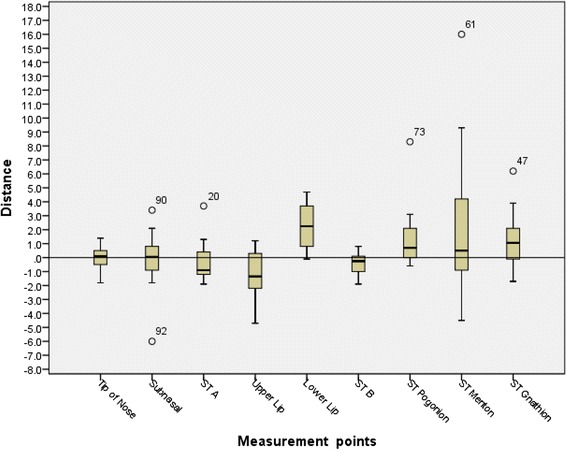
*X*-axis data box-plot

**Fig. 5:**
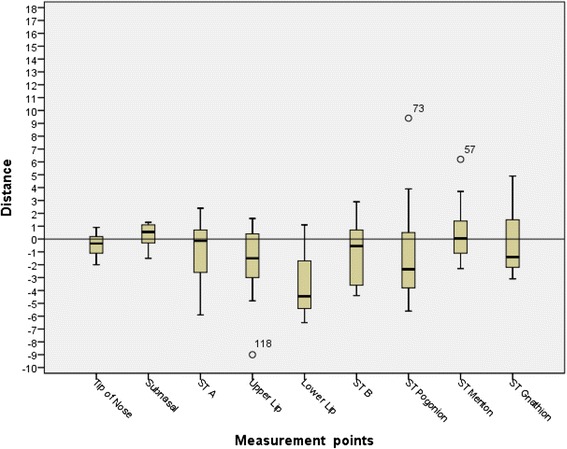
*Y*-axis data box-plot

Dolphin Imaging software version 11.0.03.37 (Patterson Dental Supply, St. Paul, MN) was utilized to perform cephalometric tracing and analysis (Table 1; Figs. 1 and 2). The cranial base was used as a reference to perform superimpositions (Fig. 3). The superimposition allowed actual changes achieved by surgery to be analyzed and recorded for each subject. The maxillary movement was recorded at ANS and A point and the mandibular movements at B point and Pg in both the *X*- and *Y*-axis (Tables 2 and 3; Figs. 4 and 5).

**Table 1:** Description of cephalometric landmarks used in this study

Hard tissue points	
Anterior nasal spine (ANS):	Anterior tip of the bony process of the maxilla
Articulare (Ar):	A point at the junction of the posterior border of the ramus and the inferior border of the posterior cranial base.
Gonion (Go):	Point on the curvature of the angle of the mandible located by bisecting the angle formed by lines tangent to the posterior ramus and inferior border of the mandible
Gnathion (Gn):	Midpoint between the anterior and inferior points of the bony chin
Menton (Me):	The lowest point on the symphyseal shadow of the mandible
Nasion (N):	Most anterior point on the frontonasal suture
Orbitale (Or):	Most inferior point on the inferior rim of the orbit
Posterior nasal spine (PNS):	Posterior tip of the hard palate
Pogonion (Pg):	Most anterior point on the chin
Porion (Po):	Most superior point on the external auditory meatus
Sella (S):	Geometric center of the pituitary fossa
Subspinale (A point):	Most posterior midline point in the concavity of the alveolar bone overlying the maxillary incisors
Supramentale (B point):	Most posterior midline point in the concavity of the alveolar bone overlying the mandibular incisors
Pterygomaxillare (PTM):	Lowest point of the pterygomaxillary fissure
Soft tissue points	
Glabella (G’):	The most prominent anterior point in the midsagittal plane of the forehead
Tip of the nose (Pr):	The most prominent or anterior point of the nose
Subnasale (Sn’):	The point at which the columella merges with the upper lip in the midsagittal plane
Soft tissue A point (A’):	The point of greatest concavity in the midline of the upper lip between subnasale and labrale superius
Upper lip/labrale superius (Ls):	A point indicated the mucocutaneous border of the upper lip
Stomion superius (Stms):	The lower most point on the vermilion of the upper lip
Stomion inferius (Stm_i_):	The uppermost point on the vermilion of the lower lip
Lower lip/labrale inferius (Li):	The median point in the lower margin of the lower membranous lip
Soft tissue B point (B’):	The point of greatest concavity in the midline of the lower lip between labrale inferius and soft tissue pogonion
Soft tissue pogonion (Pg’):	The most prominent point on the chin
Soft tissue menton (Me’):	Lowest point on the contour of the soft tissue chin
Soft tissue gnathion (Gn’):	Midpoint between the anterior and inferior points of the soft tissue chin

**Table 2:** Error of frequency of subjects and range of acceptable error in the *X*-axis

Patient name	Tip of the nose	Subnasale	ST A	Upper lip	Lower lip	ST B	ST Pg	ST Mn	ST Gn	Overall average
Subject 1	1.3	0.6	−1.1	−2.2	0.1	−0.6	0	9.3	3.3	
Subject 2	1.4	2.1	0.2	1.2	4.2	0.8	0.7	0.8	−0.1	
Subject 3	0.2	0.3	1.3	−1.2	4.7	−0.1	8.3	0.6	3.9	
Subject 4	−1.1	0.8	−1.3	−1.6	2.5	−0.3	−0.6	−0.9	−0.9	
Subject 5	−1.1	−1.4	−1	−4.7	4	−1.9	0.6	16	6.2	
Subject 6	0.1	3.4	3.7	0.3	3.7	0.5	3.1	−4.5	−1.7	
Subject 7	−1.8	−0.3	−1.9	0.4	3.2	−0.2	1.9	4.5	1.9	
Subject 8	−0.3	−6	−1.9	−1.5	0.8	−1	2.6	−0.4	1.4	
Subject 9	0.5	1.6	−0.7	−0.8	−0.1	−1.1	0	0.4	0.6	
Subject 10	0.1	−0.9	0.4	−2.5	1.9	−0.2	2.1	4.2	2.1	
Subject 11	−0.5	−0.2	−1.1	−1.5	1.1	0.6	1.7	0.2	1.3	
Subject 12	−0.1	0.6	0.4	0.6	3.6	0.1	0.7	3.5	0.6	
Subject 13	0.1	−0.8	−1.2	−3.5	0.5	−0.6	0.2	−1.9	0.8	
Subject 14	0.5	−1.8	−0.8	−0.5	2	−1.2	−0.2	−2.8	−1.6	
Mean	−0.05	−0.14	−0.36	−1.25	2.30	−0.37	1.51	2.07	1.27	
Mean (absolute value)	0.65	1.49	1.21	1.61	2.31	0.66	1.62	3.57	1.89	
SD	0.89	2.19	1.49	1.64	1.63	0.76	2.26	5.31	2.17	
Percent of acceptable error <0.5 mm	64 %	21 %	21 %	21 %	21 %	43 %	29 %	21 %	7 %	28 %
Percent of acceptable error <1 mm	64 %	57 %	36 %	36 %	29 %	71 %	57 %	43 %	36 %	48 %
Percent of acceptable error <2 mm	100 %	78 %	92 %	71 %	57 %	100 %	92 %	50 %	71 %	79 %

**Table 3:** Error of frequency of subjects and range of acceptable error in the *Y*-axis

Patient name	Tip of the nose	Subnasale	ST A	Upper lip	Lower lip	ST B	ST Pg	ST Mn	ST Gn	Overall average
Subject 1	0	0.9	0	0.8	−3.2	2.9	0.8	6.2	4.4	
Subject 2	0.5	1.1	−0.1	0.6	−3.4	−4.4	−4.4	−1.1	−2.2	
Subject 3	0.2	0.7	0.7	−0.1	−4.2	0.7	9.4	1.6	3.1	
Subject 4	−1.1	0.4	1.4	−1.8	−6.2	0	−2.5	0.5	−0.9	
Subject 5	−1.4	−1.5	−2.8	−4.8	−1.7	−1.6	−2.4	3.7	4.9	
Subject 6	0.2	1.3	−5.9	−9	−5.4	2.2	−3.8	1	−2.3	
Subject 7	−0.6	0.4	2.4	0.4	−6.2	−0.4	−1.4	1.4	−1.3	
Subject 8	−1.2	−0.9	−2.1	−2.2	−4.9	−1.3	0.5	−2.3	−1.5	
Subject 9	0.9	1.3	0.7	−3.9	−0.7	−0.7	−2.7	−1.4	−1.6	
Subject 10	−2	−0.8	−3.2	−1.8	−4.7	−3.6	−5.6	−2.1	−2.3	
Subject 11	−0.9	−0.3	−1.1	−3	0.6	0.3	3.9	0.1	1.5	
Subject 12	0.8	1	0.8	1.6	−6.5	−4	−5.2	−0.7	−3.1	
Subject 13	−0.5	0	−2.6	−0.4	1.1	1.6	0.3	−0.2	0.7	
Subject 14	−0.2	1.3	−0.2	−1.2	−5.2	−4	−2.3	0	−1.8	
Mean	−0.38	0.35	−0.86	−1.77	−3.61	−0.88	−1.10	0.48	−0.17	
Mean (absolute value)	0.75	0.85	1.71	2.26	3.86	1.98	3.23	1.59	2.26	
SD	0.87	0.92	2.23	2.79	2.53	2.40	3.98	2.29	2.64	
Percent of acceptable error <0.5 mm	43 %	29 %	21 %	36 %	0 %	21 %	14 %	29 %	0 %	21 %
Percent of acceptable error <1 mm	71 %	64 %	43 %	36 %	14 %	36 %	21 %	43 %	14 %	38 %
Percent of acceptable error <2 mm	100 %	100 %	100 %	100 %	57 %	57 %	28 %	57 %	28 %	61 %

The software was then used to superimpose the profile pictures taken pre-treatment with digitally traced soft tissue landmarks of the pre-treatment cephalometric radiograph. This “linked” both images and helped initiate the software’s VTO simulation. Dental landmarks were also traced and superimposed to account for orthodontic movements during treatment. Finally, the software generated a prediction profile photograph. This image was compared to the actual patient post-treatment profile photograph taken 6 months after surgery so that any swelling due to the surgical procedure had subsided and the soft tissue changes were stable. Soft tissue landmarks, which were identified on each photograph, were compared through superimposition to determine differences. Differences between the prediction and actual coordinates of nine soft tissue landmarks (Pr, Sn, A’, Ls, Li, B’, Pg’, Gn’, and Me’) in each axis were then calculated, tabulated, and analyzed (Tables 4 and 5). When tabulating the data, a positive value represented a more anterior position of the prediction compared to the surgical outcome and a negative value represented a more posterior position. The value allows determination if the prediction was an underestimation or overestimation of the achieved surgical result.

**Table 4:** Differences between prediction and actual coordinates in the *X*-axis

Patient name	A point	ANS	Pogonion	B point
Subject 1	3	3	−4	−4
Subject 2	3.9	4	−2.3	−3
Subject 3	1.6	1.6	−13.7	−11.5
Subject 4	1.9	0.6	−9.4	−7.8
Subject 5	1	1.9	−15.7	−11.7
Subject 6	4.4	4.4	−10.5	−10
Subject 7	1	2	−8.4	−6.5
Subject 8	3	3	−10.1	−10.3
Subject 9	1.8	2.8	−6.4	−5.3
Subject 10	1	1	−12.8	−12.1
Subject 11	1.3	0.8	−5.9	−5.1
Subject 12	1.1	0.5	−10.8	−11.2
Subject 13	3.6	3.6	−3	−2.4
Subject 14	2.9	3.4	−6.8	−5.3
Mean	2.25	2.33	−8.56	−7.59
SD	1.19	1.31	4.05	3.48

**Table 5:** Differences between prediction and actual coordinates in the *Y*-axis

Patient name	A point	ANS	Pogonion	B point
Subject 1	0.2	0.2	−0.2	−0.2
Subject 2	0.9	0.9	2.3	2.6
Subject 3	1.1	1.1	0.3	−4.3
Subject 4	0.2	1.5	3.3	6.9
Subject 5	−2.6	−1.7	−1	2.9
Subject 6	0.8	0.8	2.2	2.4
Subject 7	0	0	4.5	4.2
Subject 8	0.1	0.1	−2.4	−0.1
Subject 9	0.6	0.6	−0.7	−0.7
Subject 10	0.8	0.8	0.8	2.4
Subject 11	−1.1	−1	0	−1.5
Subject 12	2.4	0.8	1.9	3.4
Subject 13	−1.7	−1.7	−0.3	−0.5
Subject 14	0.4	0	4.8	4.8
Mean	0.15	0.17	1.11	1.59
SD	1.25	1.00	2.13	2.94

### Statistical analysis

#### Power analysis

A sample size of 12 produces a two-sided 95 % confidence interval with a margin of error of 2 mm when the estimated standard deviation is 3.000. Data was tabulated using Microsoft Excel (Version 14.1.0, Redmond, WA), entered into SPSS software (Version 21.0, Chicago, IL), and subsequently analyzed. The mean, standard deviation, and 95 % confidence interval for the difference measured at each landmark were calculated. This was done in both the *X*- and *Y*-axis. A percentage of acceptable error was calculated for landmarks with a value of +/− 0.5, 1.0, and 2.0 mm. Two-sided 95 % confidence interval is used as margin of error.

#### Measurement reliability and reproducibility

Five randomly selected patient radiographs were re-traced, VTO repeated, and re-measured twice by the same investigator (RP) and then by another investigator (RL). The above was completed at least 4 weeks after the initial tracings and VTO prediction analysis. Intra-class correlation coefficient (ICC) was used to assess both intra-examiner reliability and inter-examiner reliability, using the two-way mixed and absolute agreement model.

## Results

Intra-examiner results showed that ICCs are 0.729 and 0.834 in the *X*-axis and 0.694 and 0.533 in the *Y*-axis for the investigator RP and investigator RL, respectively, which indicated that a good reliability in the *X*-axis and moderate reliability in the *Y*-axis existed between repeated measurements of each investigator. All measurements from the investigator RP were averaged and compared to an average of the second investigator’s measurements (RL). The inter-examiner correlation coefficient was determined to be 0.747 in the *X*-axis and 0.613 in the *Y*-axis, which are in the acceptable range.

A point moved an average of 2.25 mm anteriorly 0.15 mm superiorly and ANS moved anteriorly an average of 2.33 and 0.17 mm superiorly during the surgical movements. Pg moved posteriorly an average of 8.56 and 1.11 mm superiorly while and B point an average of 7.59 and 1.59 mm superiorly.

The differences in soft tissue landmarks between the prediction and actual results were greater in the vertical rather than the anterior-posterior direction. In the sagittal direction Pr, Sn, A’, Ls, and B’ were on average a negative value (more posterior). Li, Pg’, Me’, and Gn’ were all on average a positive value (more anterior). Ls was the most posteriorly positioned value (−1.25 mm) and Li was the most anteriorly positioned value (2.30 mm). In the vertical direction, all values were inferior except Sn and Me’. Li was by far the most inferior value in the vertical direction (−3.61 mm).

In the anterior-posterior plane, Pr prediction was the most accurate landmark (0.65 mm), followed by B’ (0.66 mm). Me’ was the least accurate (3.57 mm) landmark, followed by Li (2.31 mm). In the vertical direction, the tip of the nose prediction was the most accurate measurement (0.75 mm) while the lower lip prediction was the least accurate (3.86).

The prediction error of Dolphin Imaging VTO was analyzed by tabulating the error frequency of subjects within the range of acceptable error in both the *X*-axis (Table 2) and *Y*-axis (Table 3). Three categories (0.5, 1.0, and 2.0 mm) were used to analyze the data based on increasing allowance of error. Two millimeter has been cited as the maximum error allowable before it does not have any value to the patient or clinician [5].

In the *X*-axis, Pr (64 %) and B’ (43 %) were proven to be accurate with a high frequency (error <0.5 mm). Gn’ had the least frequency of acceptable error at 7 %. When the acceptable error was 2 mm, Pr and B’ were accurate with a frequency of 100 %. Li and Me’ were the least accurate with 57 % and 50 % frequency, respectively. Judging by the confidence intervals of the mean of prediction error, we are 95 % confident that the means of prediction error of Pr, Sn, STA, and STB are within the acceptable error of 2 mm; while others may have a possibility that the mean error is over 2-mm threshold.

In the *Y*-axis Pr (43 %) and Ls (36 %) were the most frequently accurate landmarks (error <0.5 mm). Li did not have any accurate landmarks with a frequency of 0 %. Pr and Sn’ had a frequency of 100 % accuracy (error <2 mm). Li and Pg’ had the poorest accuracy (26 %). Also, the confidence intervals reveal that we can be 95 % confident that means of prediction error of Pr, Sn, STMn, and STGn are within the acceptable error of 2 mm; others may have a possibility that the mean error is over 2-mm threshold. So, we can be 95 % confident that the prediction error for Pr and Sn is within acceptable 2-mm threshold in both *X*- and *Y*-axis.

## Discussion

Understanding the accuracy of the predictions made by Dolphin VTO can help a clinician in the treatment planning of complicated surgical cases and better inform patients and set realistic expectations. It is important to consider that the VTO predictions utilized in this study assume that pre-surgical orthodontic tooth movements and surgical jaw and tooth movements would be perfectly executed as treatment planned. Actual soft tissue profile results would likely differ more significantly from the VTO predictions if the surgical procedures were not executed exactly as planned.

The Dolphin Imaging VTO calculates predictions with two separate linear parameters based on the direction of movement in the *X*- or *Y*-axis. This study revealed that Dolphin Imaging had varying degrees of accuracy at each soft tissue landmark in both the horizontal and the vertical axis. The computer predictions were more consistently accurate in the sagittal direction than the vertical direction. A much larger standard deviation was seen at almost all landmarks in the *Y* direction as compared to the *X* direction. This data differed from the finding of Lu et al., who showed the *Y* direction as being more consistently accurate with prediction results [11]. Lu suggested that this might occur because the computer generated surgery mainly involves sagittal algorithms. Our findings conflict with this statement.

We analyzed 14 patients in this study, which was higher than the 12 patients suggested by the power analysis. The results of the calculated prediction error in this study were very consistent with previous studies. Accuracy was 79 % (*X*-axis) and 61 % (*Y*-axis) with and error of acceptable error set at 2.0 mm. If Me’ and Gn’ are excluded, the accuracy increased to 84 % (*X*-axis) and 63 % (*Y*-axis). A study by Pektas et al. calculated an overall error of 91 % for the sagittal direction and 68 % in the vertical direction for errors <2.0 mm [12]. Pektas’ study however did not include Me’ and Gn’. Me’ and Gn were less accurate as compared to other landmarks in the horizontal direction.

Kazandjian et al. found that prediction errors of 1.0–2.0 mm were found to be clinically acceptable by orthodontists, surgeon, and lay people [13]. Most literature categorizes errors into <1.0-, 1.0–2.0-, and 2.0-mm groups. Although this acceptability of error 1.0–2.0 mm was found to be reliable, Kaipatur and Flores-Mir discussed that compounding areas of acceptable errors individually might lead to an overall unacceptable prediction [14]. Hence, different categories of acceptable error were calculated in this study to allow for increasing degrees of error analysis and in-depth interpretation of the results.

Gosset et al. showed that Dolphin had an even distribution of both over estimation and underestimation among tested landmark [4]. Sinclair et al. indicated an underestimation of the prediction value from lips to E plane [15]. In a study looking at bimaxillary setback surgery, it was found that Dolphin predictions tended to overestimate the amount of soft tissue retraction except for the Sn and Pg’ [11]. This study’s analysis demonstrated that the landmarks overlying the maxilla (Pr, Sn, A’, and Ls) were under estimated (negative value) of the actual soft advancement in the horizontal direction. The soft tissue landmark overlaying the mandible (Li, Pg’, Me’, and Gn’) all showed an overestimate (positive value) of the amount of actual soft tissue retraction. B’ was the only landmark that was underestimated. It is important to point out that in the current study the subjects had much larger movements of the mandible as compared to the maxilla.

In our study, Li prediction was least accurate. The lower lip is influenced by the type of skeletal malocclusion [16, 17], incisor position, angulation, soft tissue thickness, and tonicity; perioral musculature and muscle attachments [12]. The accuracy was within 2.0 mm 57 % (*X*-axis) and 14 % (*Y*-axis), respectively. This agrees with most of the literature, which showed that the lower lip is the weakest area predicted in the Dolphin VTO [18–22]. Another reason may be due to the program’s linear algorithms while in realty the lower lip may respond in a non-linear fashion. Dolphin Imaging offers an “auto lip adjustment feature,” which allows the investigator to easily change the position of the lips. This may help investigators to accommodate for the inaccuracy of the lower lip prediction.

Pr and B’ were most accurate in the *X*-axis. Both exhibited 100 % accuracy for an error less than 2 mm. The tip of the nose may be least affected by maxillary advancement (2.25 mm on average in this study). The chin is often the chief complaint of surgical patients. Pg’ prediction has been shown to be 100 % accurate within 2 mm of error [12]. We found an acceptable error of 92 %. This may be due to the shape of the human chin. Identification of the chin’s soft tissue landmarks will be most accurate on a line tangent to the chin in the horizontal or vertical direction. This may explain why Pg’ was most accurate in the *X*-axis and Gn’ and Me’ were more accurate in the *Y*-axis.

Few limitations of this study are due to the fact that this was a retrospective study and all subjects in this study were not operated by one surgeon. This may have affected the post-surgical outcome and photographs taken by several clinicians. A prospective study would have been more ideal. Although, it would be extremely hard to execute due to the complexity of these cases. The analysis of the Dolphin VTO in this study was two-dimensional. Future studies should re-analysis the sample using three-dimensional technology to compare the results.

## Conclusions

1. Dolphin Imaging software tool can be utilized for case presentation and patient education and to obtain patient’s informed consent for two-jaw orthognathic surgical treatment plans.
2. The VTO software program could be useful for gross movements and predictions of two-jaw surgical outcomes during treatment planning but is unreliable for treatment planning precise movements when measurement range is <1 mm.
3. The lower lip prediction is the least accurate in this program for two-jaw surgical cases. The use of this program must be with caution to prevent unrealistic patient expectations and dissatisfaction.
